# An insight into cancer palaeobiology: does the Mesozoic neoplasm support tissue organization field theory of tumorigenesis?

**DOI:** 10.1186/s12862-022-02098-3

**Published:** 2022-12-13

**Authors:** Dawid Surmik, Justyna Słowiak-Morkovina, Tomasz Szczygielski, Maciej Kamaszewski, Sudipta Kalita, Elżbieta M. Teschner, Dawid Dróżdż, Piotr Duda, Bruce M. Rothschild, Dorota Konietzko-Meier

**Affiliations:** 1 Institute of Earth Sciences, Faculty of Natural Sciences, University of Silesia, Będzińska 60, 41-200 Sosnowiec, Poland; 2 Institute of Paleobiology, Polish Academy of Sciences, Twarda 51/55, 00-818 Warsaw, Poland; 3 Institute of Animal Sciences, Warsaw University of Life Sciences, Ciszewskiego 8, 02-786 Warsaw, Poland; 4 Institute of Geosciences, Section Paleontology, University of Bonn, Nussallee 8, 53115 Bonn, Germany; 5 Institute of Biology, University of Opole, Oleska 22, 45-052 Opole, Poland; 6 Faculty of Exact and Technical Sciences, University of Silesia, Będzińska 39, 41-200 Sosnowiec, Poland; 7 Carnegie Museum of Natural History, 4400 Forbes Ave, Pittsburgh, PA 15215 USA

**Keywords:** Neoplasm, Mesozoic, Paleopathology, Comparative oncology

## Abstract

**Background:**

Neoplasms are common across the animal kingdom and seem to be a feature plesiomorphic for metazoans, related with an increase in somatic complexity. The fossil record of cancer complements our knowledge of the origin of neoplasms and vulnerability of various vertebrate taxa. Here, we document the first undoubted record of primary malignant bone tumour in a Mesozoic non-amniote. The diagnosed osteosarcoma developed in the vertebral intercentrum of a temnospondyl amphibian, *Metoposaurus krasiejowensis* from the Krasiejów locality, southern Poland.

**Results:**

A wide array of data collected from gross anatomy, histology, and microstructure of the affected intercentrum reveals the tumour growth dynamics and pathophysiological aspects of the neoplasm formation on the histological level. The pathological process almost exclusively pertains to the periosteal part of the bone composed from a highly vascularised tissue with lamellar matrix. The unorganised arrangement of osteocyte lacunae observed in the tissue is characteristic for bone tissue types connected with static osteogenesis, and not for lamellar bone. The neoplastic bone mimics on the structural level the fast growing fibrolamellar bone, but on the histological level develops through a novel ossification type. The physiological process of bone remodelling inside the endochondral domain continued uninterrupted across the pathology of the periosteal part.

**Conclusions:**

Based on the results, we discuss our case study’s consistence with the Tissue Organization Field Theory of tumorigenesis, which locates the causes of neoplastic transformations in disorders of tissue architecture.

**Supplementary Information:**

The online version contains supplementary material available at 10.1186/s12862-022-02098-3.

## Introduction

Investigating the pathogenesis and tumour growth dynamics in non-human animals promotes understanding of neoplasm biology and indirectly helps to develop treatment strategies for cancer in humans. Veterinary and comparative oncology document the current occurrences of neoplasms in various taxa. Examination of the occurrence of neoplastic diseases in various extinct taxa across the Phanerozoic supplements this current state of knowledge with respect to the mechanisms of tumour development in an evolutionary perspective, as well as documents the somatic vulnerability of extinct animals to neoplasms. The fossil record, although limited mainly to hard tissues, provides an exceptional insight into the tumorigenesis across the animal kingdom in the evolutionary time.

Occurrences of cancer were documented in extinct amniotes—in a Late Permian-Early Triassic therapsid [1], a Middle Triassic diapsid [2], and more comprehensively in avian [3] and non-avian dinosaurs [4–9]. The extensive amount of available dinosaur material allows for derivation of some palaeoepidemiological inferences [3, 9]. In contrast, documentation of neoplasms in non-amniotes is scarce, both in the herpetological and paleontological literature [8, 10–15]. Osseous abnormalities of unknown origin, which can be considered neoplastic, were identified in the extinct Late Cretaceous giant salamander of Uzbekistan [14]. Two alleged cases of neoplasms were also reported in temnospondyls from the Early Triassic of Russia [13, 15], but the reliability of their diagnoses is currently being questioned.

Here, we provide the first documentation of bone tumour growth dynamics and its malignancy in a vertebral intercentrum of an extinct large temnospondyl amphibian, *Metoposaurus krasiejowensis* Sulej 2002 [16] from the Late Triassic of Krasiejów in Poland.

## Methods

The specimen ZPAL Ab III/2467 is a block of rock containing cervical and anterodorsal intercentra. The cervical intercentrum is free from pathology and will not be discussed further. Due to the tight diagenetically related connection of both elements, the macrophotographs and ground-sectioning include both bones but for the purpose of 3D reconstructions the non-pathological bone was digitally removed.

The specimen was photographed using an Olympus digital camera and scanned with GE Phoenix v|tome|x, at 200 kV, 300 μA; scanning time of 1 h 17 min; resolution 70 µm; projection images were captured using a 2024 × 2024 px scintillator/CCD with an exposure time of 500 ms. The equipment is installed in the Department’s Laboratory of Microtomography, University of Silesia.

The ontogenetic growth and regular histology of *Metoposaurus* intercentra is well known [17, 18], therefore, the non-pathological intercentrum was not sampled and not considered here in detail. ZPAL Ab III/2467 was ground-sectioned according to the standard petrographical method [19]. First cut was done subhorizontally, close to the ventral margin of the pathological intercentrum, strongly obliquely, with the left cutting margin below the left parapophysis and the right margin going through the remains of the right parapophysis. That cutting plane was selected to include the largest possible surface of the pathological tissue. Due to its size, the pathological vertebra had to be placed on two separate slides: lateral right part of the pathological vertebra was separated from the rest of the specimen by an oblique, posteromedial cut. This was done after the initial, subhorizontal cut, so both parts are sectioned along the same plane. The loss of tissue due to the additional cut is minimal and the microstructural characteristics are continuous on its both sides. The surface of the entire section represents the ventral and right lateral surface. The second cut in transverse plane was done from the anatomically dorsal half of the vertebra and represent the surface between the left part of the ventral margin, left lateral side, articulation to neural arch and remains of the right parapophysis.

## Results

### Morphological description

**Fig. 1:**
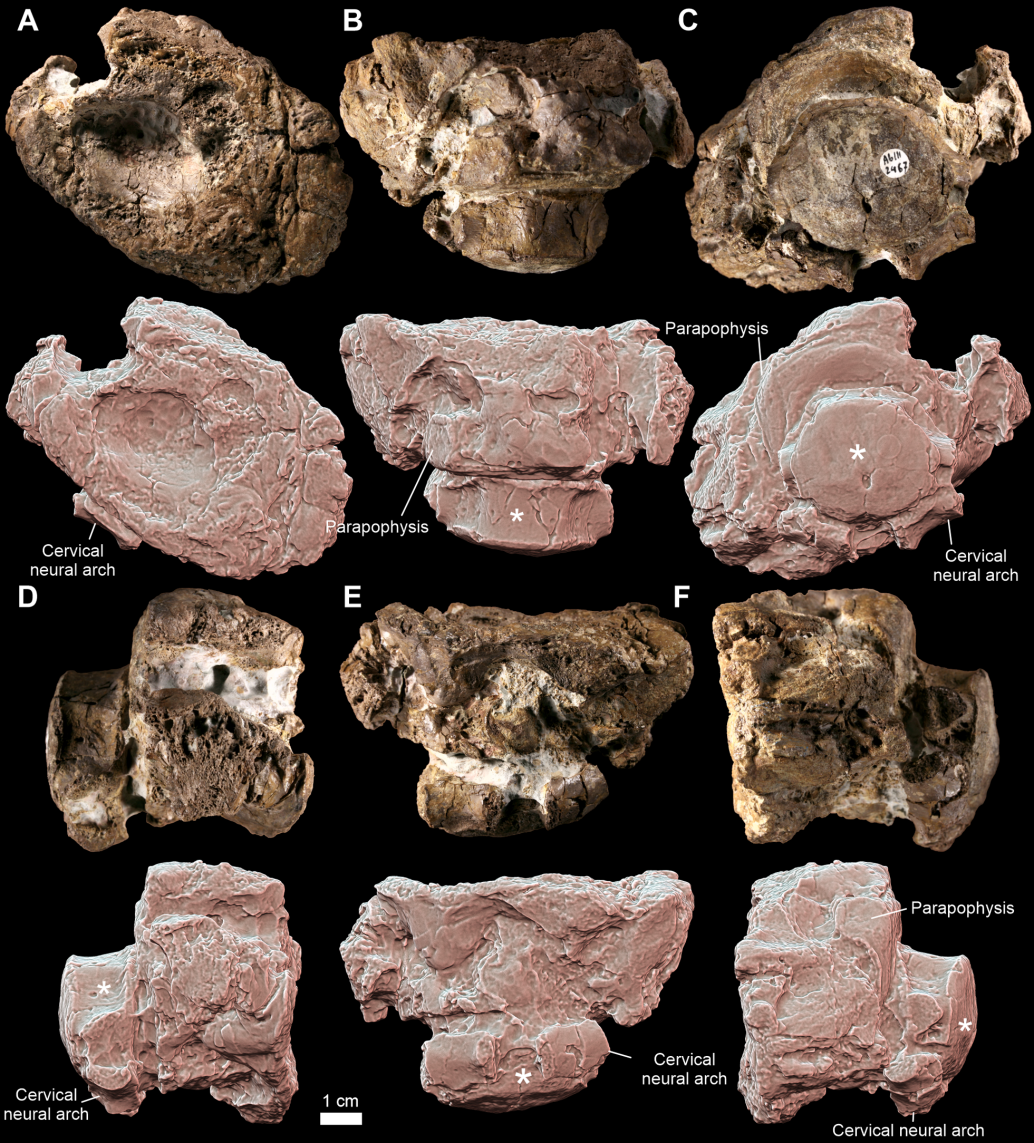
The neoplasm-affected *Metoposaurus krasiejowensis* vertebra. The photographs (upper rows) and 3D models in orthographic view with radiance scaling (lit sphere) shader enabled (lower rows) of the pathological dorsal intercentrum ZPAL Ab III/2467 in (**A**) anterior, (**B**) dorsal, (**C**) posterior, (**D**) lateral right, (**E**) ventral, and (**F**) lateral left view, and associated normal cervical centrum (asterisk). Note that the cervical intercentrum is displaced and located upside down and backwards relative to the pathological dorsal intercentrum

The specimen ZPAL Ab III/2467 (Fig. 1) consists of two independent intercentra (Fig. 1E, F, asterisk), one non-pathological and the second altered by massive pathological overgrowth. The non-pathological bone (Fig. 1) has a convex anterior and concave posterior surface, both the diapophyses and parapophyses are located close to each other and aligned in a vertical line as well as the neural arch fused with the intercentrum’s body. These characteristics are typical for the vertebrae from the cervical region of the vertebral column [18, 20]. The second intercentrum is severely pathologically malformed (Fig. 1). The only recognizable fragments are the dorsal-posterior margin and a left parapophysis (Fig. 1B, C.). Based on the shape of both structures (the parapophysis is large and longer than half the length of the intercentrum and concave posterior area), the vertebra could be identified as anterodorsal [18, 20]. Most of the posterior surface is covered by the cervical intercentrum (Fig. 1C–E) and thus not visible. The remaining part of the intercentrum is overgrown by an abnormal bony overgrowth (Fig. 1) with numerous irregular fossae, grooves and pits. The pathology on the left side is primarily located ventrolateral, below the left parapophysis; contralaterally, the right parapophysis is included into the dorsolateral overgrowth, so it is not visible in the gross examination. A particularly deep notch is present on the dorsolateral surface between the convex structure coving the region of the physiological articulation with the neural arch and the overgrowth around the right parapophysis (Fig. 1B). The anterior face of the pathological mass is flattened and centrally bears a prominent concavity encircling the anterior articular surface (Fig. 1A).

### XMT virtual sections

**Fig. 2:**
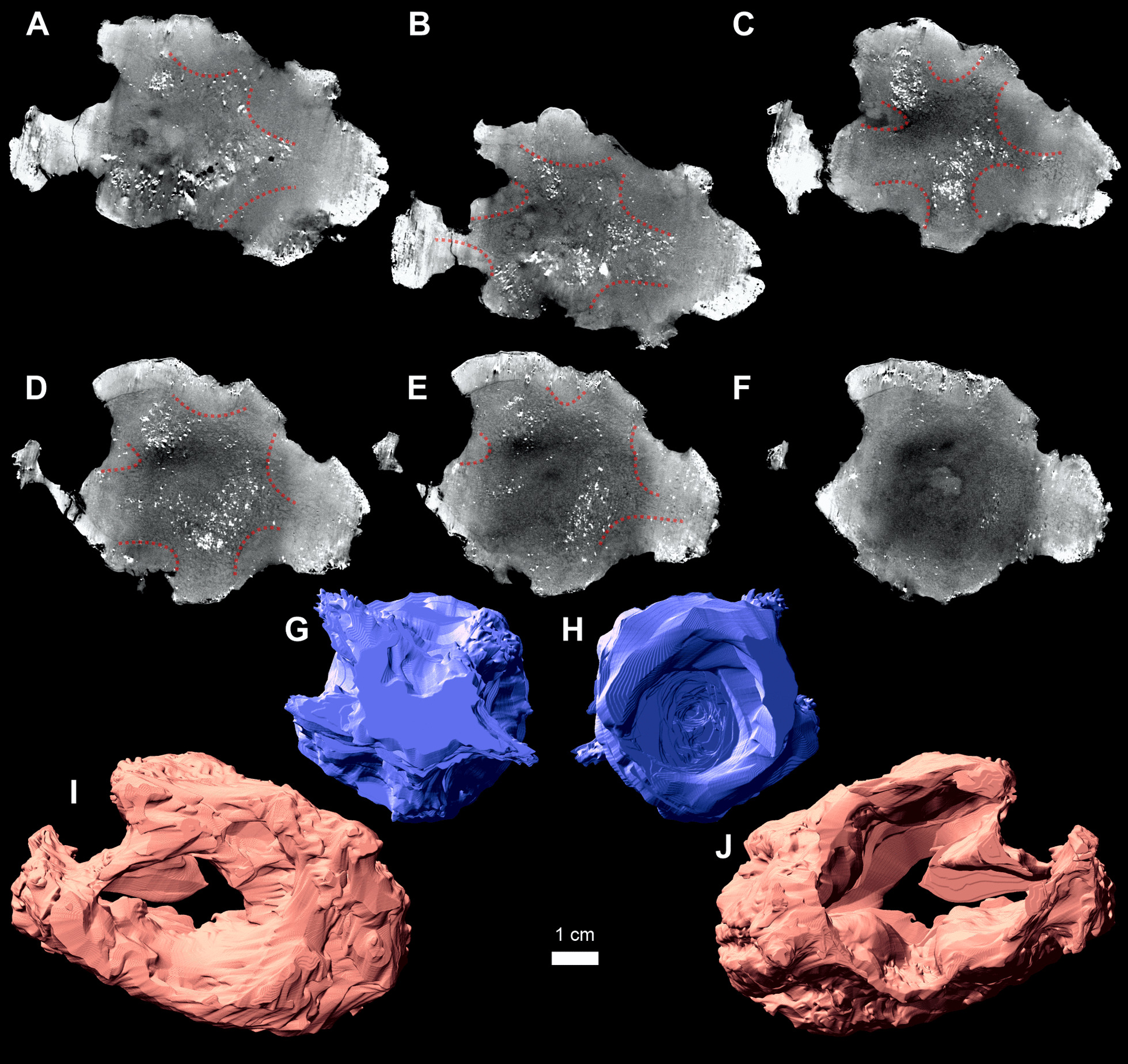
*Metoposaurus krasiejowensis* ZPAL Ab III/2467. **A**–**F** coronal sections through the pathological vertebral intercentrum (anterior towards posterior, right side towards the left side of the page) with star-shaped structure (red dotted line); **G**, **H** 3D volumetric reconstruction of the remaining normal part of the pathologically-altered intercentrum in (**G**) anterior and (**H**) posterior view exhibiting a subconical shape with star-like process visible in its anterior part and more oblate in the posterior part; **I, J** 3D reconstruction of the outgrowth in (**I**) anterior and (**J**) posterior view. The planes of virtual sectioning are presented in Additional file 5. The physical cutting planes of the specimen are consistent with the virtual sectioning shown in Additional file 5

The 3D data reveal the morphology of the studied specimen (Additional file 1), separations on the pathologically altered vertebral intercentrum (Additional file 2, blue object), and bone overgrowth (Additional file 2, coral pink object). The three-dimensional XMT digital imaging of the intercentrum reveals that the posterior surface is almost free from pathological structures, with the exception of its ventral margin (Fig. 2). Posteriorly and anteriorly to the midsection, the amount of non-pathological bone gradually decreases. The reconstruction of the inner structures reveals that normal tissue is mostly limited to a star-shaped remnant in the central part of the intercentrum, built from irregular trabeculae (Fig. 2A–F, within dotted lines). The cavities are considerably larger anteriorly (Fig. 2). Two longer and narrower arms of the star, separated by a deep notch, point dorsally and slightly dorsolaterally to the right. Two shorter and wider arms are present laterally, and the last arm points ventrally, with a delicate inclination to left (Fig. 2A–F). In the anteroposterior projection, the amount of the normal tissue decreases anteriorly which produces a conical shape with the posterior surface of the intercentrum continuing the base of the cone (Fig. 2G, H, Additional file 3).

The volume of the pathologic bone mass (Fig. 2I, J) is two times greater than the remains of the affected intercentrum (50 cm^3^: 22 cm^3^, Fig. 2G, H, see Additional files 2, 3, 4).

### Histological description

**Fig. 3:**
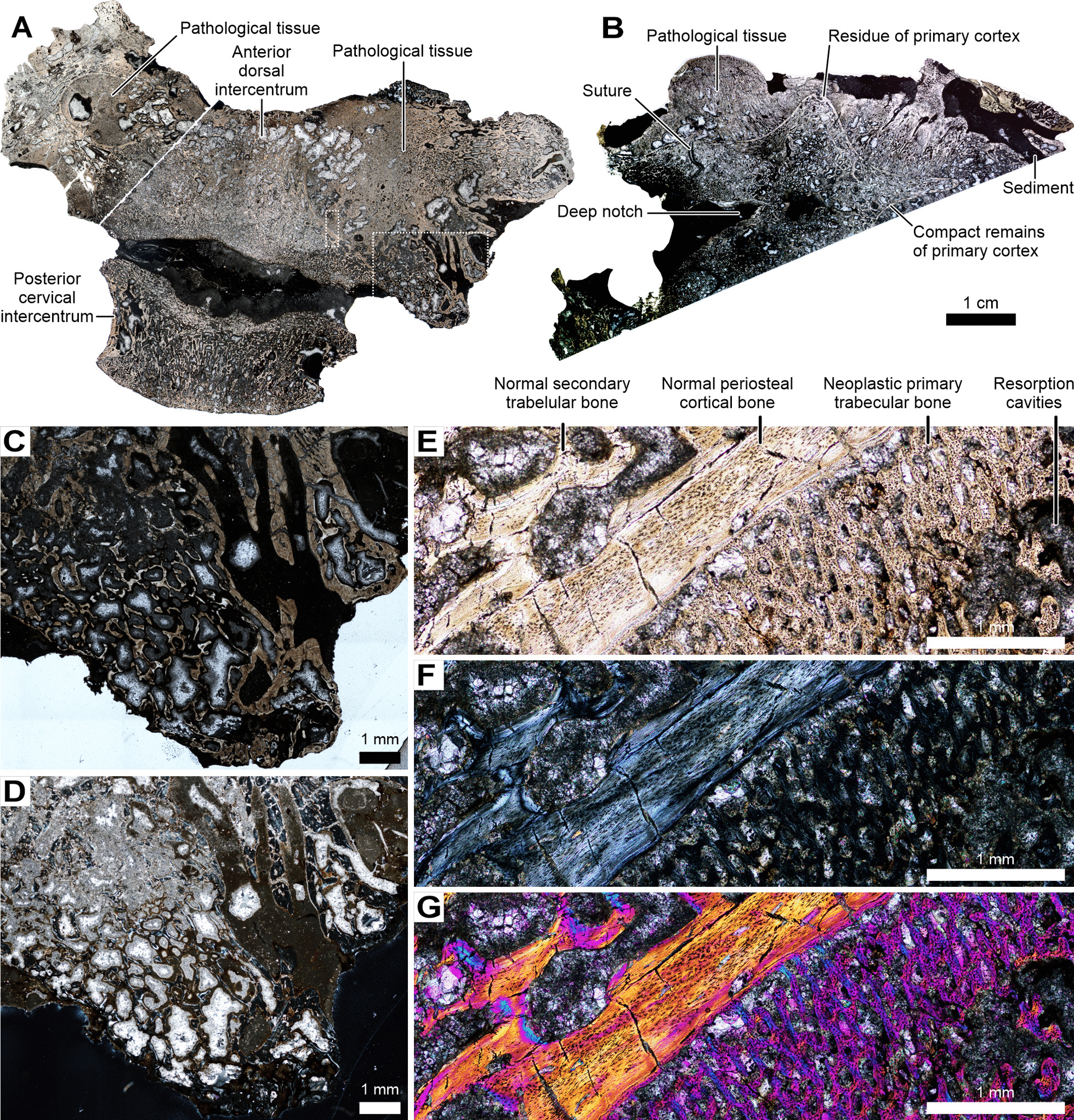
*Metoposaurus krasiejowensis* ZPAL Ab III/2467, histological sections. **A**, oblique subtransverse section through the entire specimen (pathological anterior dorsal intercentrum and normal cervical intercentrum); **B**, coronal section through the dorsal part of the pathological intercentrum; **C**, **D**, closeup of the external part of the pathology (larger rectangle in **A**) showing neoplastic (secondary?) lamellar trabecular bone with large irregular cavities in (**C**) normal transmitted and (**D**) polarized light; **E**, **F**, close-up of the frontier between normal secondary trabecular and primary periosteal cortical bone, and new trabecular bone with destructive cavities in (**E**) normal transmitted, **(F**) polarized, and (**G**) polarized light with lambda wave plate

The studied intercentrum is derived from three microstructurally different regions: first, characterised by trabecular bone with irregularly arranged trabeculae, corresponding to the endochondral domain of the anatomically normal intercentrum; second, compact remains of the periosteal cortex; and third, highly vascularized pathological tissue (Fig. 3). The entire intercentrum underwent an intensive remodelling with loss of intrinsic trabeculae resulting in a porous appearance. The remodelling is more advanced next to the anterior surface and in the central part of the bone, as well as in the externalmost parts of the bone, as visible, respectively, in the sub-horizontal (Fig. 3A) and transverse sections (Fig. 3B).

The sub-horizontal section of the pathological intercentrum (Fig. 3A) clearly shows two architectonically different histological structures. The central part of the sectioned pathological intercentrum is built from trabecular bone, separated from the new growth by a layer of the compact bone resembling the normal periosteal cortex. The line starts next to the posterior margin of the intercentrum on the left lateral aspect of the specimen, and ends anteriorly mesial next to the original anterior surface (Fig. 3A). However, the anterior fragment of the border underwent remodelling resulting in a smooth merging of the pathological and normal bone through secondary bone (Fig. 3B). A comparable remnant of periosteal bone on the right side of the intercentrum (due to the oblique cutting plane) is visible as a swollen lip-shaped line, which separates the affected tissue from the new bone and with extensive remodelling next to the anterior surface of the bone (Fig. 3).

In the transverse section the margin between the normal and pathological tissues forms a star-shaped structure. The right ventrolateral arm of the star corresponds to the notch-like structure in the affected tissue visible in the sub-horizontal section (Figs. 2, 3). The remains of the affected cortex surround the entire star-shaped structure and are partially also visible as the external most layer in the deep dorsolateral notch (Fig. 3B). Residual fragments of periosteal bone are preserved in the tips of the arms. The bone overgrowths surrounding the notch on both sides are separated by sutures from the remaining part of the intercentrum (Fig. 3B). Remains of bone fragments, separated by a layer of sediment, are visible on the left side of the section, in the region below the left parapophysis (Fig. 3).

Histologically, the remains of the normal bone tissue are represented in the endochondral domain as secondary lamellar trabeculae without any remains of calcified cartilage and in thin layers of avascular highly organized parallel-fibred tissue as remains of periosteal bone (Fig. 3E–G). Flat and moderately numerous osteocyte lacunae are visible in the innermost part of that layer. In the external layer, the number of osteocyte lacunae rapidly increases (Fig. 3E–G).

The pathological bone varies in framework from a well-organized, very highly vascularized, and regular trabecular net (Fig. 3C–E) to more compact and irregularly shaped in the excrescences (Fig. 3F, G). The primary pathological tissue deposited in the regions directly bordered by the remains of the cortex is highly vascularized with the radially-organized vascular canals. The tissue is exclusively composed of lamellar bone with only small patches of highly organized parallel-fibered bone. It hosts extremely large and numerous osteocyte lacunae, which are not organised in rows (Fig. 3E–G). In that region, some cavities are visible, surrounded by highly vascularised primary tissue. The external most part of the bone loses its regular arrangement and cavities are separated only by thin lamellar trabeculae (Fig. 3E–G).

## Discussion

### The comparison with the regular histology

The regular intercentrum is built of two domains, endochondral and periosteal. The trabeculae in the endochondral part are not organized, in contrast to periosteal bone in which the trabeculae are regularly arranged. Endochondral ossification developed first, followed by periosteal ossification, initially fast and subsequently slowing down during the ontogeny [17, 18]. The border between these two domains is usually well defined in small individuals thanks to a different orientation of trabeculae, but in older it is less visible due to remodelling, resulting in a smooth transitional zone built of secondary trabeculae. The older the bone ontogenetically, the thicker the secondary layer. Numerous calcified cartilage residua are always preserved in the primary endochondral domain, but this tissue is not related to the ontogenetic age of *Metoposaurus* [17]. The preservation of calcified cartilage extending long into the ontogeny seems to be plesiomorphic for all Stereospondyli [18].

In a transversally sectioned normal intercentrum, primary trabecular bone of the endochondral domain is almost entirely surrounded (except for the dorsal surface) by highly vascularized primary cortex, representing the periosteal domain [17, 18]. Periosteal bone visible in the normal intercentrum represents the lamellar-zonal bone tissue type with parallel-fibred matrix and numerous vascular canals organized in regular rows in zones, whereas annuli are avascular. Deeper parts of the periosteal bone are usually strongly remodelled and primary bone is visible only in a form of small patches between the cavities. A few deep canals, perforating the surface and reaching to the endosteal domain, are visible, representing probably nutrient canals [17] (Additional file 6).

In the pathological bone analysed herein, the entire inner part of the star-shaped structure in the pathological intercentrum, with the exception of the tips of its arms, is filled with secondary trabecular bone and it is not possible to determine the border between the primarily periosteal and endochondral domains. Surprisingly, the analysed specimen does not show any calcified cartilage in endosteal domain in both sections. The lack of calcified cartilage in the sub-horizontal section of the intercentrum of the analysed specimen is due to the sectioning plane, limited to the periosteal part of the affected bone. However, the lack of any remains in the transverse section is unusual. The pathological processes causing an uncontrolled deposition of the bone in the periosteal domain may have also accelerated the physiologically slower ossification rate of the endochondral domain. Thus, even if on the microstructural level the endochondral domain does not differ very much from the regular bone, the character of the cancer affected its histological picture. Remains of the non-affected cortex are limited only to a thin, avascular layer of parallel-fibred matrix, separating the neoplastic bone from the regular secondary trabecular tissue. Small fragments of residual periosteal bone are also visible in the tips of the remaining star. These residues of tissue indicate the relatively long growth of the periosteal cortex, despite the developed pathological tissue, and represent the ontogenetically youngest part of the regular structures.

### Differential diagnosis

The massive, irregular outgrowths on the metoposaur intercentrum spread in multiple directions. The absence of draining sinus tracts, defects surrounded by new bone formation (abscess) or filigree reaction rules out an infection-related bone involvement [21, 22]. No bone deformations indicative of healed fractures were present. Absence of subperiosteal bone resorption or large resorption cavities rules out hyperparathyroidism, which would also not be associated with massive new bone deposition [22, 23]. The documentation collected from gross anatomy, histology, and X-ray computed micro-tomography suggests a fast-growing bone mass (e.g., huge lacunae and higher vascularization) and abnormal new bone deposition as a result of increased cell proliferation, characteristic for neoplasia [21, 22].

The abnormal bone deposition in this case indicates that the individual was clearly afflicted with a primary neoplasm of bone. Benign bone tumours (e.g., osteoid osteoma, osteoblastoma) form spherical protuberances composed of very dense bone [21, 22] which are absent in the studied specimen. The aggressive bone destruction and massive new bone formation indicates a sarcoma of the osteosarcoma or chondrosarcoma variety. Absence of internal curly-cue or popcorn-like calcifications rules out a cartilage-derived tumour (chondrosarcoma) [22].

**Fig. 4:**
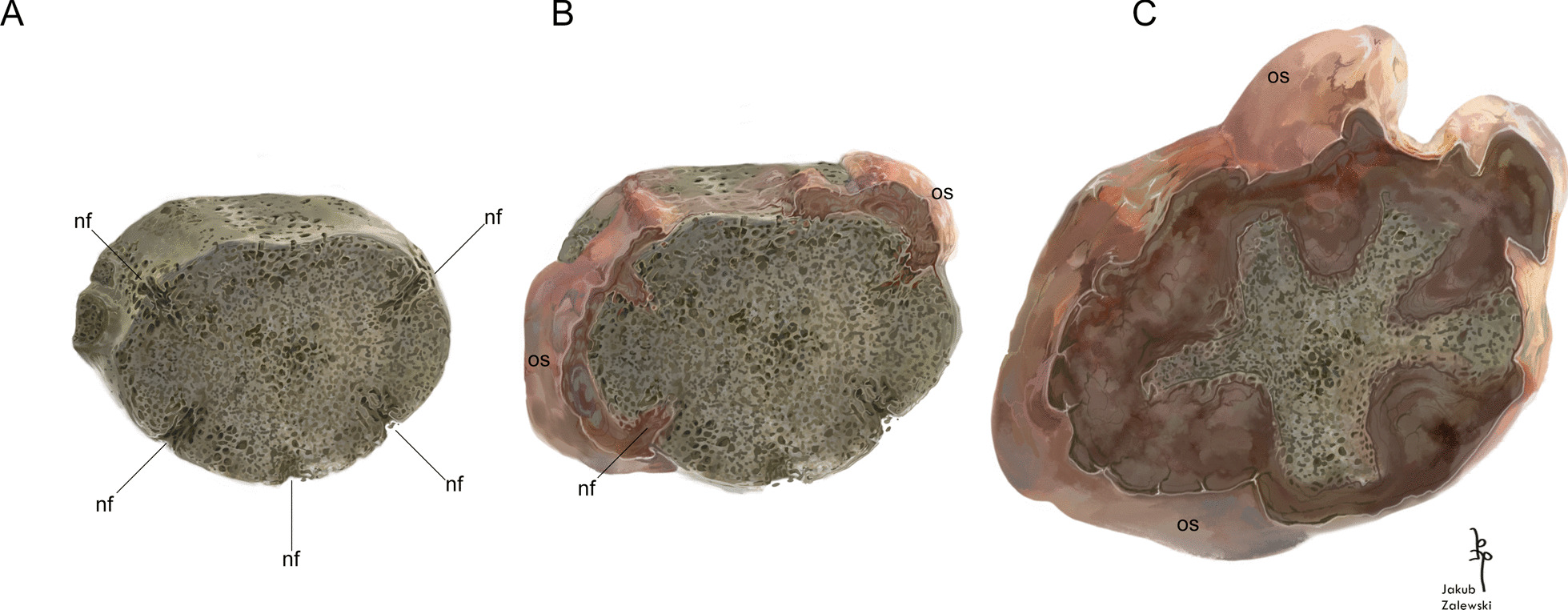
The reconstruction of growth of neoplastic tissue on the vertebral intercentrum ZPAL Ab III/2467 during ontogeny. **A** the normal, ontogenetically juvenile intercentrum, with only thin layer of the periosteal bone on the ventral part of the bone and visible nutrition foramina, **nf** reaching deep into the endochondral bone; **B** the overgrowth of the neoplasm producing osteoid, **os**, the normal periosteal and neoplasm are deposited at the same time; **C** during the growth the faster growing neoplasm gradually limit the space for normal bone. Finally, the neoplasm overgrowth the entire intercentrum. Art work by Jakub Zalewski

Osteosarcoma is the of the most common primary neoplasms affecting musculoskeletal system [24], characterized by proliferation of malignant cells of mesenchymal origin. Spinal osteosarcomas are rare and aggressive neoplasms [25]. There are different variations of osteosarcoma: central periosteal, parosteal, or telangiectatic. The latter is refuted because of absence of large osteolucent areas related to telangiectatic blood vessels [22, 26]. The location of the bone mass surrounding the affected vertebra and the clear boundary between normal and altered bone suggest that the neoplasm originated from the periosteum, rather than being centrally-derived. Further, the osteosarcoma in this individual developed from the surface of the bone (rather than within the periosteum that occurs with periosteal osteosarcoma [22]) and penetrated into the vertebral intercentrum though natural canals [17] forming the star-shape of the remaining intercentrum (Fig. 4), thus identifying it as a parosteal osteosarcoma [7, 24].

### Osteosarcoma in paleontological record

The evolutionally history of osteosarcoma is poorly understood [7]. The fossil record provides several cases of osteosarcomas in amniotes—an early Pleistocene hominid [27], a Late Jurassic dinosaur [28], and a Middle Triassic stem-turtle [2]. The identification of osteosarcoma in a Late Cretaceous ceratopsid [7] is questionable since the mentioned in its description small unconnected islands with circular morphology are not typical of osteosarcoma and more suggestive of a cartilaginous component of sarcoma, a chondrosarcoma [21, 22]. The oldest report suggesting osteosarcomatous involvement is an abnormality of integumentary cranial bones in an Early Triassic capitosaurid [13] from Russia. However, the data presented suggest extra bone pieces, typically found in Wormian bone overgrowth [29], without identifying the destructive changes characteristic of osteosarcoma [21, 22]. Since the case studied here, there is no known obvious record of osteosarcoma in extinct amphibian.

### Tumour growth dynamics

Three-dimensional analysis and distribution of the pathological tissues allow reconstruction of the dynamic of cancer growth in during its ontogeny. The morphology and the XMT visualisation reveal that the maximal development of the neoplasm is asymmetric, so the maximal deformations are present below the left parapophysis and around the right one, probably including even the region of the neural arch connection. Posterior surface of the intercentrum is the least affected by the overgrowth, whereas the anterior surface is strongly deformed. This suggests the entrance of the neoplasm from the anterior side. The latter shows extensive alterations in the deep parts of neoplasm, confirming the ontogenetically older stage of the tissue [30]. The malignant process resulted in loss of star shape visibility in the transverse section next to the anterior surface. The size of numerous areas of trabecular destruction-derived cavities in the pathological tissue exceed these known from normal tissue [17] and are typical for osteosarcoma-related bone lesions.

The most interesting structure visible in both digital and thin sections is the star-shaped structure. Histological thin sections show clearly that the characteristic for tumour, fast growing tissue is separated from the inner trabeculae by remains of the cortex. Crucial for the spread of the pathological tissue seem to be five nutrient foramina penetrating the periosteal domain of the affected intercentrum (compare Fig. 4). The comparison of the arms and the position of the canals in non-pathological bone reveals that the arms fit well between the canals, whereas the concavities are located in the regions where the canals occurred. Moreover, the almost continuous structure of the periosteal remains suggests that the neoplasm minimally permeated the endochondral domain, mostly deforming the periosteal bone. With time, the pathological tissue started to develop next to the anterior surface, overgrowing ventrally and laterally. The nutrient canals constitute a natural way for invasion of the neoplasm into the cortex. Pathological tissue was deposited rapidly as indicated by the structure of the tissue. Normal deposition was still possible in the not yet affected fragments of the periosteal bone, located between the canals, and the periosteal cortex increased its thickness. During ontogeny, both processes occurred in parallel, but the neoplasm grew faster and limited the space where periosteal growth was still possible to constantly narrowing wedges. The huge amount of osteocyte lacunae and the radial orientation of vascular canals confirm the much faster growth of the pathological tissue than the normal tissue, where only moderate number of osteocyte lacunae and considerable lower porosity are observed [17]. In the final stage, the neoplasm overgrowth surrounded almost the entire intercentrum and the physiological periosteal growth was no longer possible.

Neoplastic bone deposited close to the original vertebral centrum is well ordered. The orderliness decreases, and sinuses and cavities appear as one proceeds further from the intercentrum. The presence of other structures such as ribs of soft tissues may have limited the growth of the bone tissue. Small structures visible in the ground section in the left lateral overgrowth, clearly separated from the pathological tissue by sediment (Fig. 3B), seem to be remains of anatomically nearby-located bones such as ribs.

The notch visible in the dorsal part of the section is intriguing. Normal *Metoposaurus* spp. intercentra from the post-cervical or anterodorsal regions are not permanently fused with the neural arch [17, 31], and even in the histological sections no sign of articulation or co-ossification of both elements is visible [18, 20]. Here, the clear sutures show that in the dorsal bone mass, other elements have been included and pathologically co-ossified. The location of the notch and its shape suggest that in that case two rami of the base of the neural arch were permanently fused with the intercentrum, similar as it is in the atlas. In the case described here of the pathological condition, the notch may represent the neural canal [17, 31].

### Physiological aspects of bone tumour formation

In mammals, bone neoplasms are formed as woven bone [32], which is a fast growing matrix type resulting from the static osteogenesis [33]. Characteristic for that tissue is an unorganised structure of collagen fibres and unordered osteocytes lacunae. The studied neoplastic tissue is highly organized with numerous osteocyte lacunae. The disordered arrangement of osteocytes observed in the neoplastic bone points to static osteogenesis [33], which is connected with the formation of fast-growing woven bone. Surprisingly, in the studied metoposaur, an orderly arrangement of osteocytes lacunae is noted [33]. As a result of static osteogenesis, a bone with a high degree of packing is produced, which seems to be a structural novelty. A characteristic feature of the studied bone tumour in the metoposaur is that it is macroscopically similar to that known in amniotes, but it is differently at the level of histology. The unordered arrangement of the osteocyte lacunae can result from the very fast maturation process of osteoblasts, which mature before they are able to transit to the bone surface, as it occurs in the normal dynamic osteogenesis [33]. The dominant tissue types known from healthy bones of *Metoposaurus* are parallel-fibred or lamellar bone, both resulting from dynamic osteogenesis [18], typically found in bones that exhibit incipient fibro-lamellar bone [34]. However, even if the incipient woven bone was produced, the overall tissue was not as highly vascularised and rich in osteocyte lacunae, and the overall growth rate was not as high as observed in the mammalian neoplastic bone [32]. It is possible that genetic limitations of the organism made it impossible to produce fast growing true woven bone and it was metabolically more effective and only possible to accelerate the growth rate via increasing the number of osteocyte lacunae, their maturation rate, and extremely high vascularisation. However, it is not clear how the scattered bone cells were able to produce a highly organised tissue. The huge amount of osteocyte lacunae and the radial orientation of vascular canals confirm the much faster growth of the pathological tissue than the normal tissue, where only a moderate number of osteocyte lacunae and considerably lower porosity are observed [17]. Large and subspherical osteocyte lacunae (Additional file 7) were noted in pathological states in fossils [35–37]. However, there are no explanations of the role of these osteocytes in cancer biology known from paleohistological studies. Recent medical studies have shown that direct attack by cancer cells on osteoblasts induces the less-organized osteoblast arrangement [38] and proved that osteocytes as important components of the cancer microenvironment in the bone where cancer cells alter osteocyte viability and their gene expression profile [39, 40]. Thus, osteoblasts which in a physiological state deposit highly organized collagen fibres and established on the bone surface regular rows of osteocytes [33], mature earlier in the bone matrix before shifting to the bone surface and deposit bone in an abnormal fashion, still lamellar on the collagen fibres level but disorganized structurally, enormous porous with a high number of primary trabeculae. The last character seems to be crucial for the estimation of bone growth rate. The fact that the tumour finally overgrowth the entire healthy tissue and limited its growth indicates that pathological tissue was growing faster than the healthy one, however only in a relative way. It is not possible to state how long it takes for the neoplasm to develop to the size observed here. Despite the diagenetic alteration and destruction, the pathologic process is clearly visible in the examined specimen. Wherever the tumour had a space to grow, it grew in an orderly manner (blood vessels arranged radially); where there was an obstacle, the tumour bone lost its organization.

It is, therefore, noteworthy that while no woven bone is observed in ZPAL Ab III/2467 and the pathological region is composed of well-organized, lamellar matrix accompanied by high number of radial vascular canals, the tissue is rich in subspherical (regardless of the sectioning plane) osteocyte lacunae and relatively loosely arranged spatially trabeculae, with the axes locally oriented predominantly outwardly, in a sense mimicking rapidly deposited radial fibrolamellar bone tissue of amniotes [41, 42]. This suggests that while on one hand the growth rate was limited by the deposition rate of the tissue determined by its organized weave, on the other hand, that was compensated by its spatial layout. While radial (or spicule-like) deposition of periosteal bone is known to occur in pathological scenarios [37, 43], no comparable tissue type was thus far described in Mesozoic amphibians.

### Theories on aetiology of cancer

Neoplasms are common across the animal kingdom and affect most vertebrates [8, 44] constituting a part of class heritage. Tumorigenesis seems to be plesiomorphic for animals—spontaneous tumours appear even in basal metazoans [45]. As metazoans exhibit a natural propensity to proliferate [46], the clonal cell divisions are controlled by genetic mechanisms. In the conventional, currently accepted paradigm, called Somatic Mutation Theory (SMT) [47], development of neoplasm is related with the loss of genetically-conditioned coordination of cell proliferation and differentiation [48]. It is a consequence of evolution, development of clonal multicellularity, and increase of somatic complexity. According to this paradigm, the transformation of a normal cell into a neoplastic cell occurs directly through DNA damage and any cell in the organism can become cancerous. Hypothetically, it means that the greater the somatic complexity of the organism, the more susceptible it should be to cancer. However, this is not true, since in large animals such as pachyderms [49], the prevalence of cancer is lower than in small mammals. This phenomenon is known as Peto’s paradox [50] and is one of big challenges in modern comparative oncology.

The somatic mutation paradigm is prevalent and sets the directions for current cancer research. In 1999, Sonnenschein & Soto [51] proposed an alternative theory, in which tumorigenesis takes place when the normal interaction between the functional calls (parenchyma) and structural cells (stroma) of organism is disturbed. According to that view, neoplasm should be examined from a hierarchical perspective of the organism and defined as a problem of tissue organization. This theory was named Tissue Organization Field Theory (TOFT) [47], because it recognizes that the primary source of cancer is the loss of organization at the tissue level, not genetic mutations [47]. Cells in an organism’s tissues undergo numerous processes that regulate their metabolism. A number of intercellular communication mechanisms which guarantee a correct organization and coordination of cells at every stage of ontogenesis, including histogenesis, are responsible for the regulation of tissue physiology. Disruption of communication processes, related, for example, with the state of cell membrane polarization and disruption of ion transport through ion channels and ion gaps, may consequently lead to changes in the organization and functioning of tissues [52]. Such changes may include: (1) disorders of tissue metabolism, (2) increase in cell proliferation and mobility, (3) transformation of mature cells into stem cells, which in turn may lead to the development of neoplasms. Both theories describing carcinogenesis have been widely studied and discussed. Although SMT and TOFT describe two different mechanisms of cancer formation, there are attempts to combine the two theories. However, as suggested by Montévil and Pocheville [53] because of the differences in the logical assumptions of these theories, they should not be combined.

**Fig. 5:**
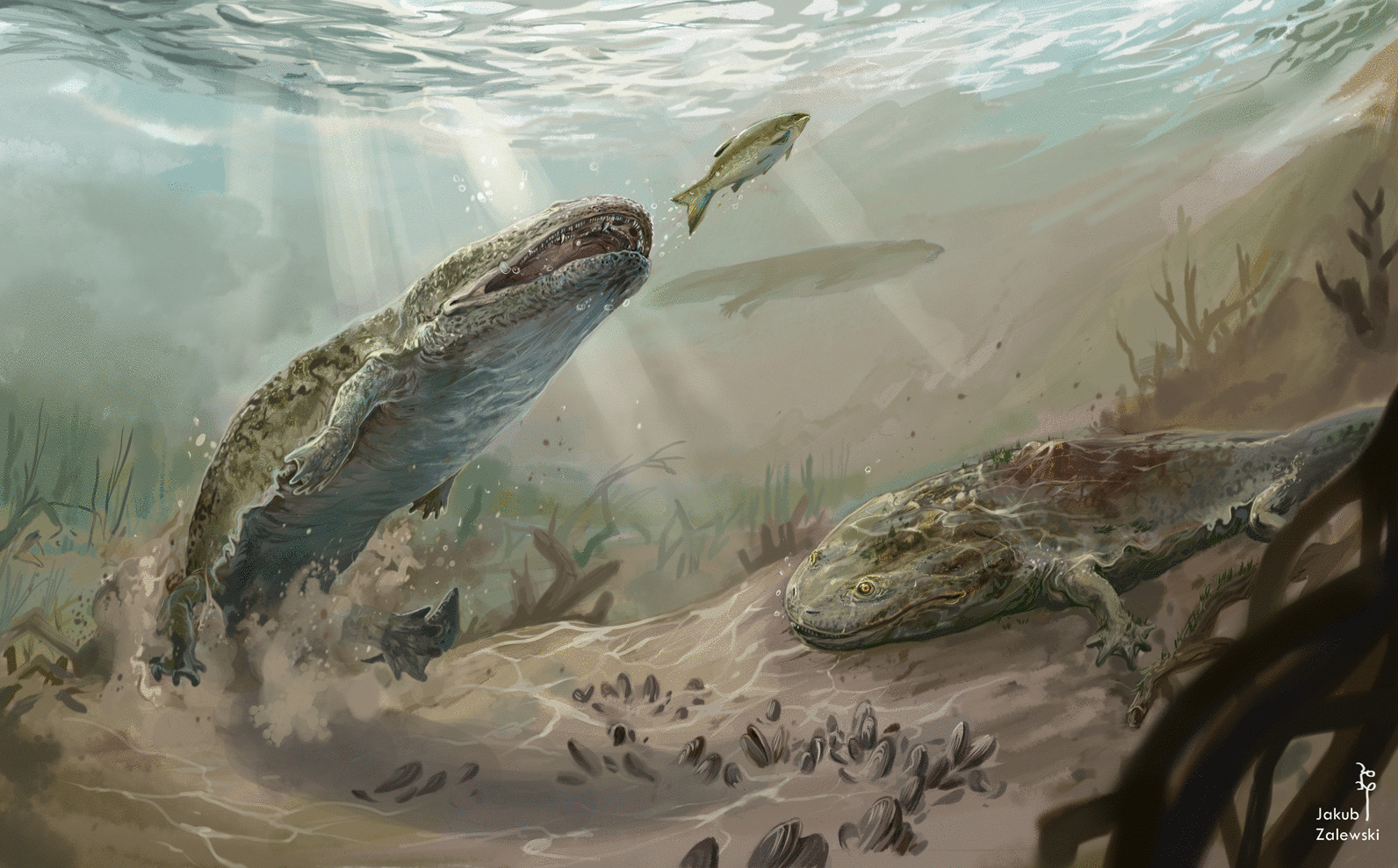
The life reconstruction of metoposaurs in their environment. The neoplasm-affected individual (on right), which the specimen ZPAL Ab III/2467 is representing. In artistic reconstruction, the affected individual has a prominent tumour in the anterodorsal region of spine. It has limited mobility and hunting activity, appears to be malnourished. Art work by Jakub Zalewski

The discussed case of cancer in a metoposaur (Fig. 5) is consistent with the Tissue Organization Field Theory, which locates the causes of neoplastic transformations in disorders of tissue architecture. This is expressed in several ways: (1) the fast growth characteristics of the newly formed bone which mixes a slowly deposited matrix type with spatial distribution typical for rapidly growing bone; (2) both the affected intercentrum and the overgrowth being subject to physiological remodelling processes, as evidenced by the numerous areas of bone tissue destruction within the tumour and the vertebra itself; it appears that the physiological processes occur in the neoplasm and the original bone alike; (3) it is difficult to explain why the remains of the cortex exhibited as the star-shaped structure are so well marked; in case of a typical neoplastic invasion, lesions and a chaotic organisation could be expected but in the described specimen the border between the physiological bone and the overgrowth is ordered and clear. In the analysed bone a change of tissue state by an increase of its cell proliferation and its subsequent hypertrophy can be observed, which led to the modification of the epigenetic state of its cells into a stem-like state.

Amphibians, thanks to their developmental and ecological plasticity and high regeneration potential, are a vertebrate group in which neoplasms are rarely described. As the amphibians go through the process of metamorphosis, during which the organism undergoes a reconstruction, it is assumed that they have mechanisms protecting them from carcinogenesis and point mutations within oncogenes. Because of that, they are an object of research in the area of both comparative and clinical oncology, as well as an aid in search of new strategies of neoplasm therapies and are utilized in research focused on understanding of potential mechanisms of anti-neoplasm defence. Ruben et al. [54] indicate that the mechanisms involved in the amphibian metamorphosis may also protect the organism from neoplasms. On the other hand, the rarity of identification and description of neoplasms in amphibians may also result from the issue of poor general recognition of pathology in that group [55]. A literature review of 50 cases of neoplasia in exotic amphibians (between 1954 and 2018) revealed that the most common neoplasms, mostly concern skin [12]. The resistance for cancer in some bigger mammals like pachyderms [56] and whales [57] is now recognized that an increase in repeated representation of a specific gene provides protection against cancer development [58] and was developed during a natural selection in the course of evolution [57, 58]. Molecular sequencing indicate the remote antiquity of the tumour suppressor family genes [59, 60]. A genome duplication and production of tetraploids happen in the course of amphibians evolution [61], so the duplications of tumour suppressor genes also can occur.

## Conclusion

The studied specimen is intriguing because it documents advanced bone cancer in the extinct relative of group of tetrapods which is believed to be resistant for cancer. It provides a well-documented case of osteosarcoma, a rare bone neoplasm and its presence in the Late Triassic. Furthermore, the growth dynamics and development of the tumour present some features which nest within the framework of TOFT for carcinogenesis.

We recommend paying special attention to bone abnormalities which could bear potential neoplasm character while examine vertebrate fossils in paleontological collections around the globe. All single reports of possible recognition of neoplasm in extinct forms should fall within the field of interest of comparative oncology.

The presented case of Late Triassic bone neoplasm expands our knowledge on occurrence of cancer in the prehistoric non-amniotes, and shows how a tumour has developed in an individual. As well in Krasiejów locality as other fossil sites of the same age, significant amounts of temnospondyl bones were found, but cases of alleged neoplasms are rare, and insufficient for neoplastic attribution so far. This may indicate that temnospondyls, like modern-day amphibians, were mostly resistant to cancer. Examining of fossilised vertebrate remains in the term of neoplasm occurrence will be helpful the create new theories that better describe the pathophysiological aspects of carcinogenesis, which further may help to develop new treatment strategies for cancer.

## Supplementary Information

**Additional file 1.** 3D model of ZPAL Ab III/2467.

**Additional file 2.** Juxtaposition of the 3D model of ZPAL Ab III/2467 and 3D volumetric reconstructions of the remaining normal part of the pathologically-altered intercentrum and bone outgrowth (tumour).

**Additional file 3.** 3D model of pathologically-altered intercentrum, virtually extracted from microtomographic scans.

**Additional file 4.** 3D model of bone outgrowth, virtually extracted from microtomographic scans.

**Additional file 5. **3D model of ZPAL Ab III/2467 with marked scanning planes.

**Additional file 6. **Photoscan of ground section of normal, non-altered anterodorsal vertebral intercentrum of *Metoposaurus krasiejowensis*, for comparative purposes, specimen number UOPB 00118 [1].

**Additional file 7.** Close-ups of osteocytes lacunae in the neoplasm-affected part of vertebral intercentrum showing their subspherical shapes.

## Data Availability

All data generated or analysed during this study are included in this published article and its supplementary information files.

## Abbreviation

ZPAL: Institute of Paleobiology Polish Academy of Sciences Warsaw Poland
