# Supplementary figures and images for: An insight into cancer palaeobiology: does the Mesozoic neoplasm support tissue organization field theory of tumorigenesis?
Source: BMC Ecol Evol. 2022 Dec 13;22:143. doi: 10.1186/s12862-022-02098-3 (PMC9746082; doi:10.1186/s12862-022-02098-3)

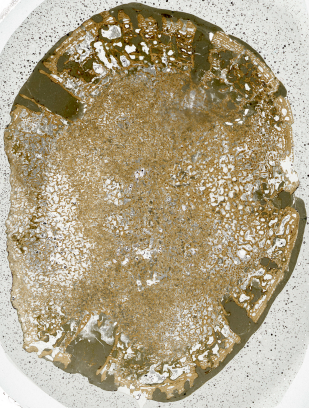

Supplement: Supplementary file 6 — Additional file 6. Photoscan of ground section of normal, non-altered anterodorsal vertebral intercentrum of Metoposaurus krasiejowensis, for comparative purposes, specimen number UOPB 00118 [1]. [file 12862_2022_2098_MOESM6_ESM.pdf]

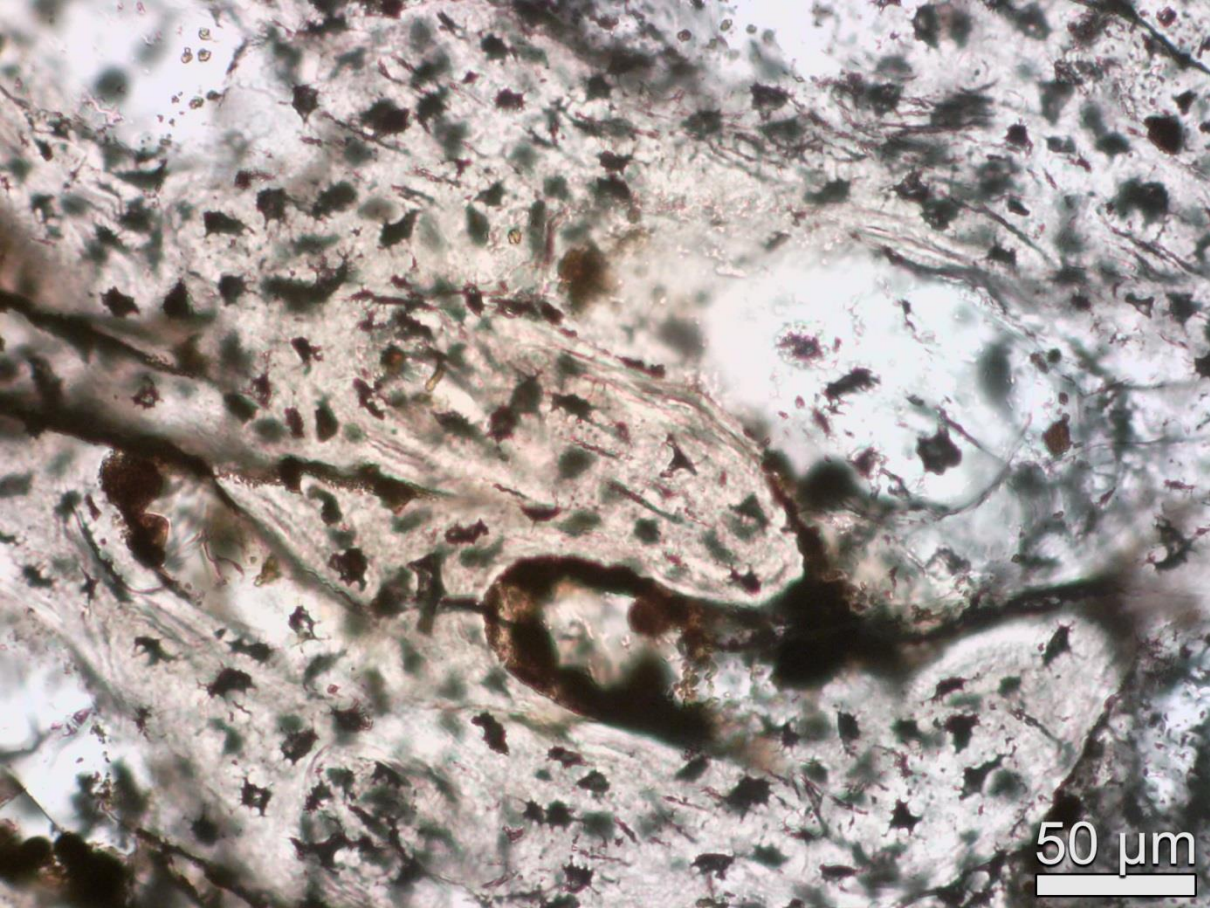

50  $\mu\text{m}$

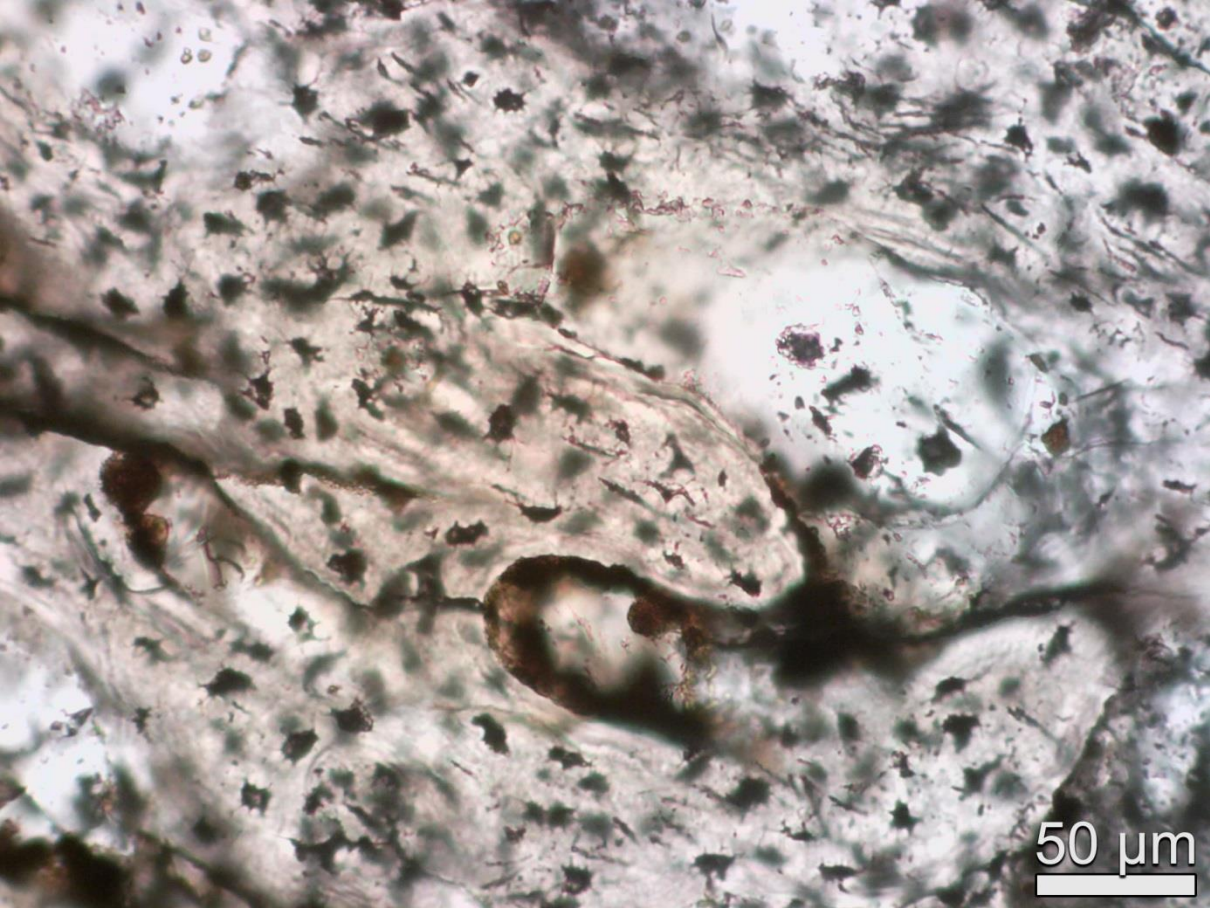

50  $\mu\text{m}$

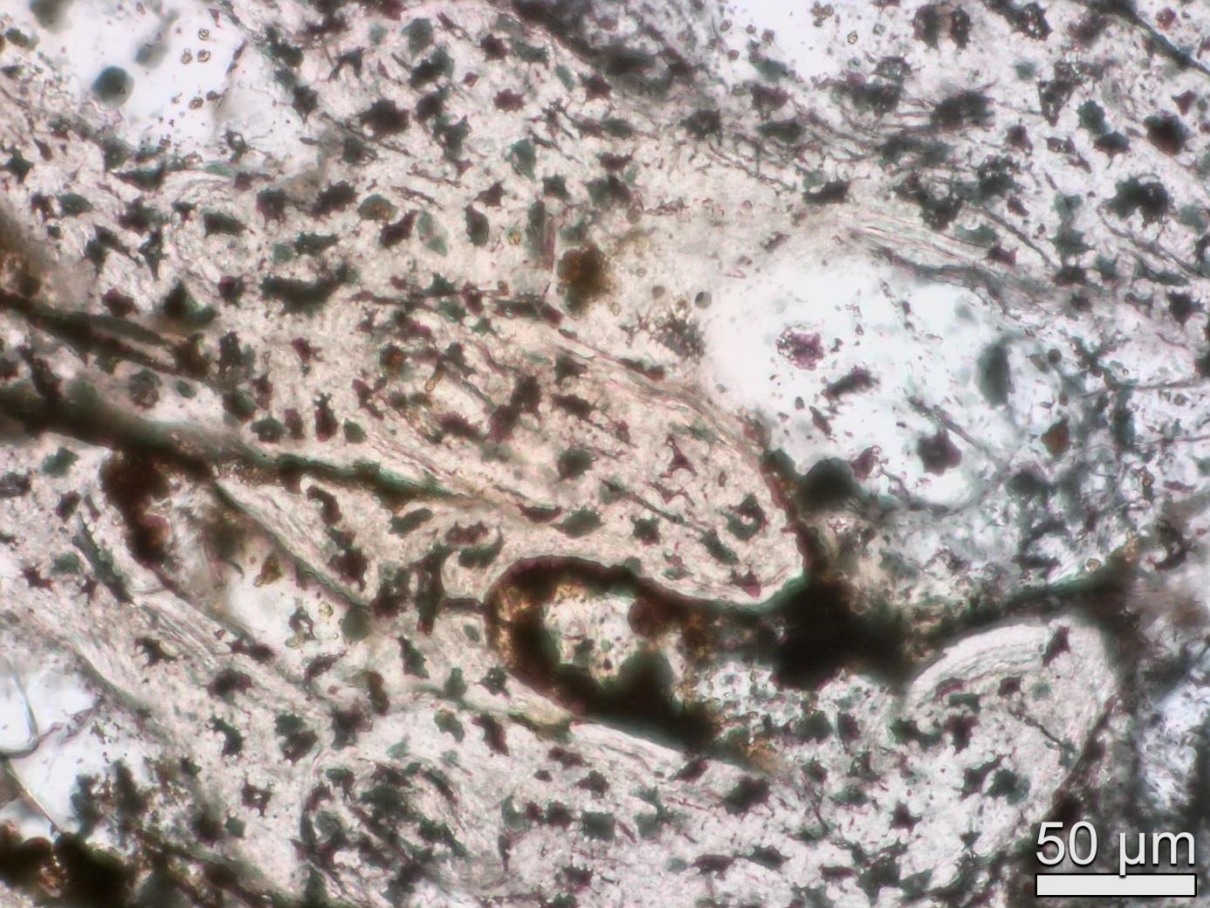

50  $\mu\text{m}$

Supplement: Supplementary file 7 — Additional file 7. Close-ups of osteocytes lacunae in the neoplasm-affected part of vertebral intercentrum showing their subspherical shapes. [file 12862_2022_2098_MOESM7_ESM.pdf]
